# Hypoxia-Induced miR-378a-3p Inhibits Osteosarcoma Invasion and Epithelial-to-Mesenchymal Transition *via BYSL* Regulation

**DOI:** 10.3389/fgene.2021.804952

**Published:** 2022-01-28

**Authors:** Junlei Zhang, Haijun Tang, Xiaohong Jiang, Nenggan Huang, Qingjun Wei

**Affiliations:** ^1^ Department of Orthopedics, The First Affiliated Hospital of Guangxi Medical University, Nanning, China; ^2^ Department of Orthopedics, Affiliated Minzu Hospital of Guangxi Medical University, Nanning, China

**Keywords:** *BYSL*, osteosarcoma, hypoxia, Nrf2, MiR-378a-3p, epithelial-to-mesenchymal transition

## Abstract

The bystin-like (*BYSL*) gene is expressed in a wide range of eukaryotes and is closely associated with tumor progression. However, its function and mechanism in osteosarcoma remain unclear. Herein, the protein expression and clinical role of *BYSL* in human osteosarcoma tissues were assessed. High expression of *BYSL* was positively related to the metastasis status and poor patient prognosis. Mechanistically, upregulation of *BYSL* enhanced Nrf2 expression under hypoxia in osteosarcoma cells. MicroRNAs are important epigenetic regulators of osteosarcoma development. Noteworthy, bioinformatics analysis, dual-luciferase reporter and rescue assays showed that miR-378a-3p inhibited *BYSL* expression by binding to its 3′-untranslated region. Analysis of miR-378a-3p function under hypoxia and normoxia showed that its upregulation suppressed osteosarcoma cells invasion and inhibited epithelial-to-mesenchymal transition by suppressing *BYSL*. Collectively, the results show that the miR-378a-3p/BYSL may associate with metastasis risk in osteosarcoma.

## Introduction

Osteosarcoma is a common malignant bone tumor, with highly invasive and systemic metastasis, that usually occurs in children and young adults (Raymond and Jaffe, 2009; Sevelda et al., 2015). Owing to new adjuvant chemotherapy and improved surgical treatment, the overall survival rate of five-years for osteosarcoma patients has improved up to 70%, while the survival rate of patients with metastasis is still only 10–30% (Ando et al., 2013; Harrison et al., 2018). Several therapeutic approaches have been developed for osteosarcoma in recent years (Li et al., 2021), but their therapeutic effects remain unsatisfactory. In-depth investigations on the molecular mechanisms underlying osteosarcoma metastasis are meaningful for the research of novel treatment approaches. Epithelial-to-mesenchymal transition (EMT) promotes the transformation of early tumors into aggressive malignant tumors, especially during tumor metastasis and invasion, which is an important event in the malignant transformation of cancer cells. EMT is characterized by a complex dynamic change, with a concomitant decline of epithelial cell markers (including β-catenin and E-cadherin) and increased mesenchymal markers (including vimentin and N-cadherin) (Arias, 2001), which has been shown to contribute to cancer metastasis and invasion in osteosarcoma (Buddingh et al., 2011; Wang et al., 2017). Therefore, inhibition of EMT is a suitable therapeutic strategy for preventing osteosarcoma metastasis.

Low oxygen tension state in tissues, known as hypoxia, has become an important factor in tumor pathophysiology. It has been reported that hypoxia triggers EMT in several types of cancers, including oral, nasopharyngeal, and gastric carcinoma (Joseph et al., 2018; Xing et al., 2021). A hypoxic environment is also intimately linked to the invasion and EMT of osteosarcoma (Shi et al., 2020). Bystin-like (*BYSL*) is a protein containing 306 amino acids encoded by the BYSL gene located on chromosome 6p21.1. It is an essential protein for embryo survival, has been identified as a sensitive biomarker for reactive astrocytes induced by ischemia/reperfusion (Aoki et al., 2006; Olczak et al., 2018). It was reported that BYSL plays a role in the biogenesis of the 40S ribosome and cell proliferation by analyzing the expression of BYSL in mouse embryos (Adachi et al., 2007). Generally, BYSL was considered to be involved in cell adhesion and growth, especially in metamorphosis (Aoki et al., 2006). Previous observations demonstrated that *BYSL* is crucial for hepatocellular carcinoma development (Wang et al., 2009). Furthermore, *BYSL* enhances glioblastoma cell migration, invasion, and EMT by controlling the GSK-3β/β-catenin pathway (Sha et al., 2020). However, the clinical role and molecular mechanism of *BYSL* in osteosarcoma metastasis remain unclear.

MicroRNAs are non-coding RNAs (17–23 nucleotides long) that, in combination with the 3′-untranslated region (3′-UTR) of the target mRNA, take part in various biological and pathological processes. MicroRNAs induce translational inhibition and mRNA degradation (Bartel, 2004), playing a critical role in cancer biology due to their involvement in the processes of proliferation, metastasis, and apoptosis (Liu et al., 2020). Therefore, miRNA profiling can be used as a biomarker for cancer prognosis and diagnosis (Lee and Dutta, 2009). Several studies have documented a strong link between miR-378a-3p function and cancer pathogenesis (Pan et al., 2019; Zheng et al., 2020). For example, overexpression of miR-378a-3p was reported to increase ovarian cancer cell development (Chanjiao et al., 2021). Moreover, breast cancer patients with low expression of miR-378a-3p in cancer tissues who were under tamoxifen treatment were found to have a weak prognosis (Ikeda et al., 2015). However, the essential significance of miR-378a-3p in osteosarcoma progression has not yet been uncovered. In the present study, the roles of *BYSL* and miR-378a-3p in osteosarcoma cell lines were examined, and their functional relationship was assessed.

## Materials and Methods

### Bioinformatics Analysis

The Gene Expression Omnibus (GEO) dataset GSE126209, comprising 12 osteosarcoma tissues and 11 paratumor tissues, was used to examine the expression of *BYSL* in osteosarcoma. We then performed bioinformatics analyses using The Cancer Genome Atlas (TCGA) sarcoma dataset to evaluate the prognostic value of *BYSL*.

### Patients and Specimens

Tissue specimens from 51 patients with conventional osteosarcoma between March 2011 and December 2019, were retrospectively analyzed after formalin fixation and paraffin embedding. Clinical data were collected from the medical records of each patient. Follow-up procedures were conducted for all patients at least once every 2 years, which included plain film, computed tomography, and magnetic resonance imaging. Further, paraffin sections were collected for this study. Based on the pathological diagnostic criteria, the pathological diagnoses were made by two pathologists. All patients with osteosarcoma agreed to participate in the study and provided informed consent. This experimental research was approved by the Institutional Review Committee of the First Affiliated Hospital of Guangxi Medical University (Nanning, China) (approval number: 2021 KY-E-041).

### Immunohistochemical Assay

Tissue samples were dewaxed by dipping in dewaxing solution and 95% ethanol, and then heated in a microwave oven to achieve antigen recovery. Following blocking with 5% goat serum, an anti-BYSL polyclonal antibody (1:300, Novus Biologicals, Littleton, CO, United States) was used for immunohistochemical analysis. Tissue specimens were incubated with BYSL antibody at 4°C for 8 h and a secondary antibody for 60 min at room temperature. Immunoreactions were visualized using diaminobenzidine for 5 min. At least 100 tumor cells were detected, using light microscopy at a magnification of 200× and 400×, in five tissue regions where the anti-BYSL antibody showed the strongest immune response. According to the level of *BYSL* expression, patients were divided into two groups—those with high expression and those with low expression. *BYSL* positivity was assessed independently by two pathologists. Immunohistochemistry results were evaluated using a scoring system as previously described (Gorlick et al., 2014). The final score, which was the product of *BYSL* positivity rate and staining intensity, was classified as low *BYSL* (0–4 points) or high *BYSL* expression (>4 points).

### Cell Culture and Transfection

Human osteosarcoma cell lines including MG63 and Saos-2 cells, obtained from the National Collection of Authenticated Cultures (Shanghai, China), were routinely cultured in Dulbecco’s Modified Eagle medium with 10% fetal bovine serum (FBS; Gibco, Waltham, MA, United States) plus 1% penicillin-streptomycin and McCoy’s 5A medium (Gibco) with 15% FBS and 1% penicillin-streptomycin, respectively. For hypoxic culture, the cells were exposed to 1% oxygen tension (1% O_2_) in a hypoxia incubator chamber. Then, miR-378a-3p-mimic, miR-378a-3p-inhibitor, *BYSL* overexpression vector (oe-BYSL), siRNA against *BYSL* (si-*BYSL*), siRNA against *Nrf2* (si-*Nrf2*)*,* and empty vector or relevant negative control (GenePharma, Shanghai, China) were transfected into the MG63 and Saos-2 cells. Transfection experiments in our study were performed using Effectene Transfection Reagent (Qiagen, Hilden, Germany).

### Luciferase Reporter Assay

The target microRNA of *BYSL* was confirmed by luciferase reporter assay, wherein the wild-type (WT, 5′-cau​cUG​UGG​CUC​CCA​GUC​CAG​g-3′) or mutant (MUT, 5′-cau​cUG​UGG​CUC​CCA​GAA​TTG​g-3′) 3′-UTR of BYSL was integrated into the pmirGLO vector (Promega, Madison, WI, United States). Cells that were cotransfected with WT or MUT *BYSL* and miR-378a-3p mimics were collected after 2 days to measure luciferase activity via a dual-luciferase reporter assay system (Promega).

### Cell Viability Assay

To assess cell viability of the experimental and control groups, we use a Cell Counting Kit-8 (CCK-8/WST-8) assay (Solarbio, Beijing, China) to detect the optical density value. Briefly, the seeding of MG63 cells was conducted in a 96-well plate and incubated for 24, 48, and 72 h. Subsequently, CCK-8 reagent was put, and the 20-min incubation of reaction mixture was performed at room temperature. The relative viability of the cells was measured at 450 nm.

### Flow Cytometry Analysis

The analysis of cell apoptosis was performed *via* flow cytometry using an apoptosis detection kit (Multi Sciences, Hangzhou, China) on the basis of the manufacturer’s specification. Cells in early and late periods of apoptosis were screened and evaluated using a Beckman Coulter CytoFLEX Flow Cytometer (Beckman Coulter, Brea, CA, United States), and FlowJo v10 software (Becton, Dickinson and Company, Franklin Lakes, NJ, United States) was adopted to analyze outcome information.

### RNA Analysis

Total RNA was extracted from cultured cells using Rneasy Mini kits (Qiagen) based on the manufacturer’s protocol. Next, cDNA was synthesized using the PrimeScript RT Master Mix cDNA synthesis system (Takara, Kusatsu, Japan), and SYBR Green (Bio-Rad, Hercules, CA, United States) was used for quantification. Expression of the gene of interest was normalized to *U6* or *GAPDH* levels. Supplementary Table S1 shows the primers applied for quantitative reverse transcription polymerase chain reaction (qRT-PCR).

### Western Blot

Proteins were extracted from MG63 or Saos-2 cells using lysis buffer (Solarbio), and the process of transferring and incubating were performed on the basis of the manufacturer’s protocols. Protein concentration was quantified using a BCA kit (Takara Bio, Inc.). The proteins were separated via SDS-PAGE on 8% gel and then electroblotted onto a PVDF membrane. Following blocking with 5% blocking reagent at room temperature for 1 h, the membranes were incubated with the primary antibodies in 5% BSA overnight at 4°C. Subsequently, the membranes were incubated with secondary antibodies at room temperature for 1 h. Proteins were visualized using ECL reagent. The primary antibodies use were the following: anti-BYSL (1:1,000, NBP1-89501; Novus Biologicals), anti-Bcl-2 (1:1,000, ab196495; Abcam, Cambridge, United Kingdom), anti-Histone H3 (1:1,000, ab18521; Abcam), anti-Bax (1:2000, ab32503; Abcam), anti-E-cadherin (1:5,000, 20874-1-AP; Proteintech, Rosemont, IL, United States), anti-N-cadherin (1:1,000, ab245117; Abcam), anti-Nrf2 (1:1,000, ab137550; Abcam), anti-β-actin (1:1,000, AC026, Abclonal), and ani-vimentin (1:2,000, 10366-1-AP; Proteintech).

### Cell Motility Analysis

To test cell migration ability, we performed wound healing assays to evaluate the migration area of cells. Briefly, after the cell density reached 100%, the cells were scratched with a fine tip and then washed with a serum-free medium to remove the unattached cells. The wound healing rate was measured by photography using an inverted microscope. For cell invasion ability, we performed transwell invasion assays to calculate invasive cells rate using transwell chambers with Matrigel (Thermo Fisher Scientific, Waltham, MA, United States).

### Osteosarcoma Subcutaneous Tumor Model in Nude Mice

MG63 cells were transfected with lentivirus stably overexpressing miR-378a-3p (miR-378a-3p-OE), and a stable cell line was established using puromycin selection. Ten male BALB/c nude mice aged 4–6 weeks were housed in the Animal Experimental Center of Guangxi Medical University. The Animal Care and Ethics Committee of the Guangxi Medical University (Number: 202108002) approved all protocols. To generate an osteosarcoma tumor *via* miR-378a-3p overexpression, MG63 cells were injected subcutaneously into the right armpit region of the mice. The animals were euthanized 25 days after cell implantation. Tumors were excised, weighed, and photographed. Tumor size in each mouse was evaluated applying the formula: V (mm^3^) = 1/6 π × length (mm) × width^2^ (mm^2^).

### Statistical Analysis

Data were analyzed using SPSS 23.0 software (IBM Corp., Armonk, NY, United States) or R software (Version 3.6.3). Univariate and multivariate analyses using the Cox regression model were performed to identify independent risk factors for osteosarcoma that influenced the total survival rate. Two or more groups were compared using Student’s t-test or one-way analysis of variance followed by Tukey’s post-hoc test, respectively. Differences were regarded as significant at *p* < 0.05.

## Results

### *BYSL* Expression is Associated With Survival Using Bioinformatics Analysis

**FIGURE 1:**
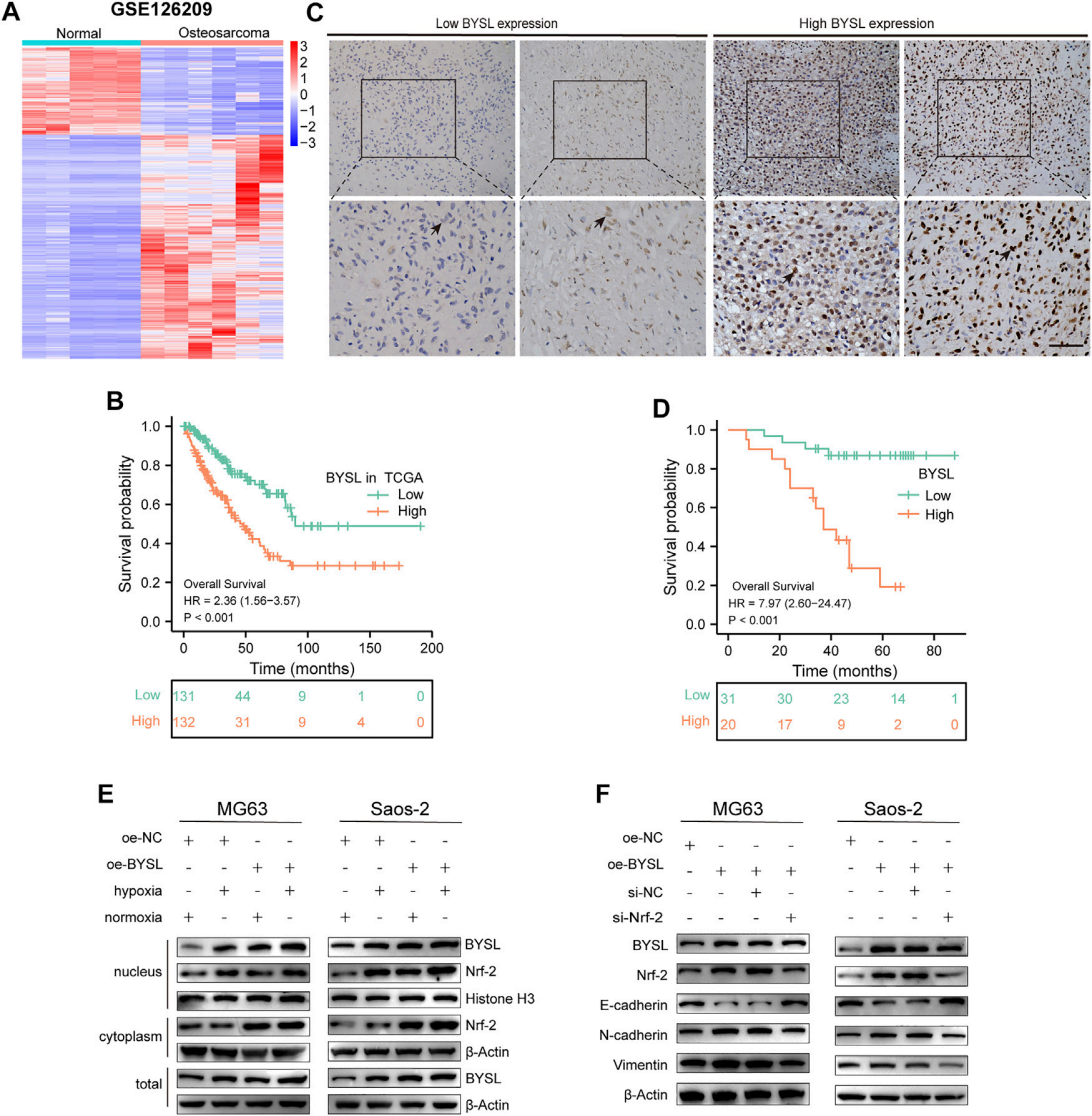
*BYSL* is related to the poor prognosis of patients with osteosarcoma. **(A)** Cluster heatmap of differentially expressed genes in GSE126209. **(B)** Overall survival was compared between osteosarcoma patients with high and low *BYSL* expression in TCGA database. **(C)** Immunocytochemical staining of *BYSL* in osteosarcoma tissues (*n* = 51, scale bar = 50 µm). **(D)** Overall survival was compared between osteosarcoma patients with high and low *BYSL* expression in an in-house cohort. **(E)** MG63 and Saos-2 cells were transfected with the control plasmid (oe-NC) or BYSL overexpression plasmid (oe-BYSL), and then cultured under hypoxic or normoxic conditions. After nuclear and cytosolic separation, protein levels of Nrf2, BYSL, Histone H3, and β-Actin were measured by western blot. **(F)** MG63 and Saos-2 cells were transfected with control plasmid (oe-NC), BYSL overexpression plasmid (oe-BYSL), si-control (si-NC), or si-Nrf2, and then cultured under hypoxic condtions. The protein levels of Nrf2, E-cadherin, N-cadheirn, Vimentin, and β-Actin were measured by western blot.

In this study, we used the GSE126209 dataset from the GEO database to evaluate *BYSL* level using the *limma* package in R. Osteosarcoma tissues were found to have higher *BYSL* expression than adjacent paratumor tissues (Figure 1A). Next, the prognostic value of *BYSL* in TCGA database was explored. Since there was no clinical data on osteosarcoma in TCGA, the sarcoma dataset was selected. Kaplan-Meier analysis showed a more adverse prognosis in patients with higher *BYSL* expression (Figure 1B).

### High *BYSL* Expression is Correlated With Poor Prognosis of Patients With Osteosarcoma

To further identify the role of *BYSL* in osteosarcoma, we first examined its expression in tissue samples of 51 patients with osteosarcoma through histopathological analysis (Figure 1C). In 51 cases of osteosarcoma tissues, BYSL was highly expressed in 20 cases and lowly expressed in 31 cases (Table 1). Next, the association between clinicopathological characteristics of the osteosarcoma patients and tissue *BYSL* expression was analyzed. Overall, high *BSYL* expression was strongly correlated with tumor-node-metastasis (TNM) stage, relapse, and metastasis (Table 1). In addition, Kaplan-Meier analysis showed a longer survival period in patients with low *BYSL* expression than in those with high *BYSL* expression (*p* < 0.01; Figure 1D). Moreover, multivariate Cox regression analysis indicated that *BYSL* and metastasis are independent risk factors for the total survival rate of patients with osteosarcoma (Table 2). Taken together, these data suggest that *BYSL* has considerable clinical significance in the prognosis and metastasis of patients with osteosarcoma.

**TABLE 1:** Associations between clinicopathological characteristics and BYSL expression in patients with osteosarcoma.

Variable	Number of patients	BYSL expression		*p* Value
		High	Low	
Age				
>16 years	24 (47.1%)	11 (55.0%)	13 (41.9%)	0.532
≤16 years	27 (52.9%)	9 (45.0%)	18 (58.1%)	—
Gender				
Female	21 (41.2%)	5 (25.0%)	16 (51.6%)	0.111
Male	30 (58.8%)	15 (75.0%)	15 (48.4%)	—
Relapse				
Yes	15 (29.4%)	11 (55.0%)	4 (12.9%)	**0.004**
No	36 (70.6%)	9 (45.0%)	27 (87.1%)	—
Metastasis				
Yes	21 (41.2%)	13 (65.0%)	8 (25.8%)	**0.013**
No	30 (58.8%)	7 (35.0%)	23 (74.2%)	—
TNM stage				
Ⅰ	29 (56.9%)	7 (35.0%)	22 (71.0%)	**0.025**
Ⅱ/Ⅲ	22 (43.1%)	13 (65.0%)	9 (29.0%)	—
Location				
Femur/Tibia	42 (82.4%)	15 (75.0%)	27 (87.1%)	0.465
Else	9 (17.6%)	5 (25.0%)	4 (12.9%)	—
Tumor size				
≤6 cm	24 (47.1%)	7 (35.0%)	17 (54.8%)	0.272
>6 cm	27 (52.9%)	13 (65.0%)	14 (45.2%)	—

Note: Bold values indicate p < 0.05.

**TABLE 2:** Univariable and multivariable Cox regression analysis of clinical characteristics and BYSL in osteosarcoma.

Variable	Univariate analysis			Multivariate analysis		
	HR	95%CI	*p* value	HR	95%CI	*p* value
BYSL	7.97	2.6–24.47	**0.00029**	6.25	1.17–33.36	**0.032**
High	—	—	—	—	—	—
Low	—	—	—	—	—	—
Age	1.81	0.7–4.69	0.2197	—	—	—
>16 years	—	—	—	—	—	—
≤16 years	—	—	—	—	—	—
Gender	0.75	0.3–1.91	0.54794	—	—	—
Female	—	—	—	—	—	—
Male	—	—	—	—	—	—
Relapse	6.02	2.25–16.13	**0.00036**	2.17	0.48–9.93	0.316
Yes	—	—	—	—	—	—
No	—	—	—	—	—	—
Metastasis	17.08	3.9–74.76	**0.00016**	30.29	2.39–383.43	**0.008**
Yes	—	—	—	—	—	—
No	—	—	—	—	—	—
TNM stage	0.11	0.03–0.39	**0.00056**	3.49	0.41–29.43	0.25
Ⅰ	—	—	—	—	—	—
Ⅱ/Ⅲ	—	—	—	—	—	—
Location	0.5	0.18–1.43	0.19659	—	—	—
Femur/Tibia	—	—	—	—	—	—
Else	—	—	—	—	—	—
Tumor size	0.37	0.13–1.05	0.06074	—	—	—
>6 cm	—	—	—	—	—	—
≤6 cm	—	—	—	—	—	—

Note: Bold values indicate p < 0.05.

### *BYSL* Overexpression Enhances Nrf2 Signaling

Next, western blot was conducted to investigate the pathway of how BYSL modulates EMT in osteosarcoma cells. Hypoxia activated the Nrf2 signaling, which has been shown to play an important role in regulation of EMT in cancer cells. Therefore, we investigated whether BYSL expression affects Nrf2 signaling. The results showed that hypoxia enhanced Nrf2 localization within the nucleus, but did not dramatically affect Nrf2 expression in the cytoplasm, indicating that hypoxia activated Nrf2 signaling. It was also found that hypoxia increased the nuclear and total expression of BYSL in osteosarcoma cells. Furthermore, *BYSL* overexpression promoted Nrf2 expression in both nuclear and extranuclear under hypoxic and normoxic conditions (Figure 1E; Supplementary Figure S1). As shown in Figure 1F, BYSL overexpression decreased epithelial marker (E-cadherin) and increased mesenchymal marker (N-cadherin and Vimentin) of osteosarcoma cells in hypoxia. However, *Nrf2* knockdown by siRNA reduced the effect of *BYSL* on EMT markers (Figure 1F; Supplementary Figure S2). The downregulation of Nrf2 did not affect the expression of BYSL, indicating BYSL was possibly upstream of Nrf2. Together, these results indicate that *BYSL* regulates the EMT of osteosarcoma cells via Nrf2 signaling under hypoxic conditions.

### miR-378a-3p Directly Targets BYSL

**FIGURE 2:**
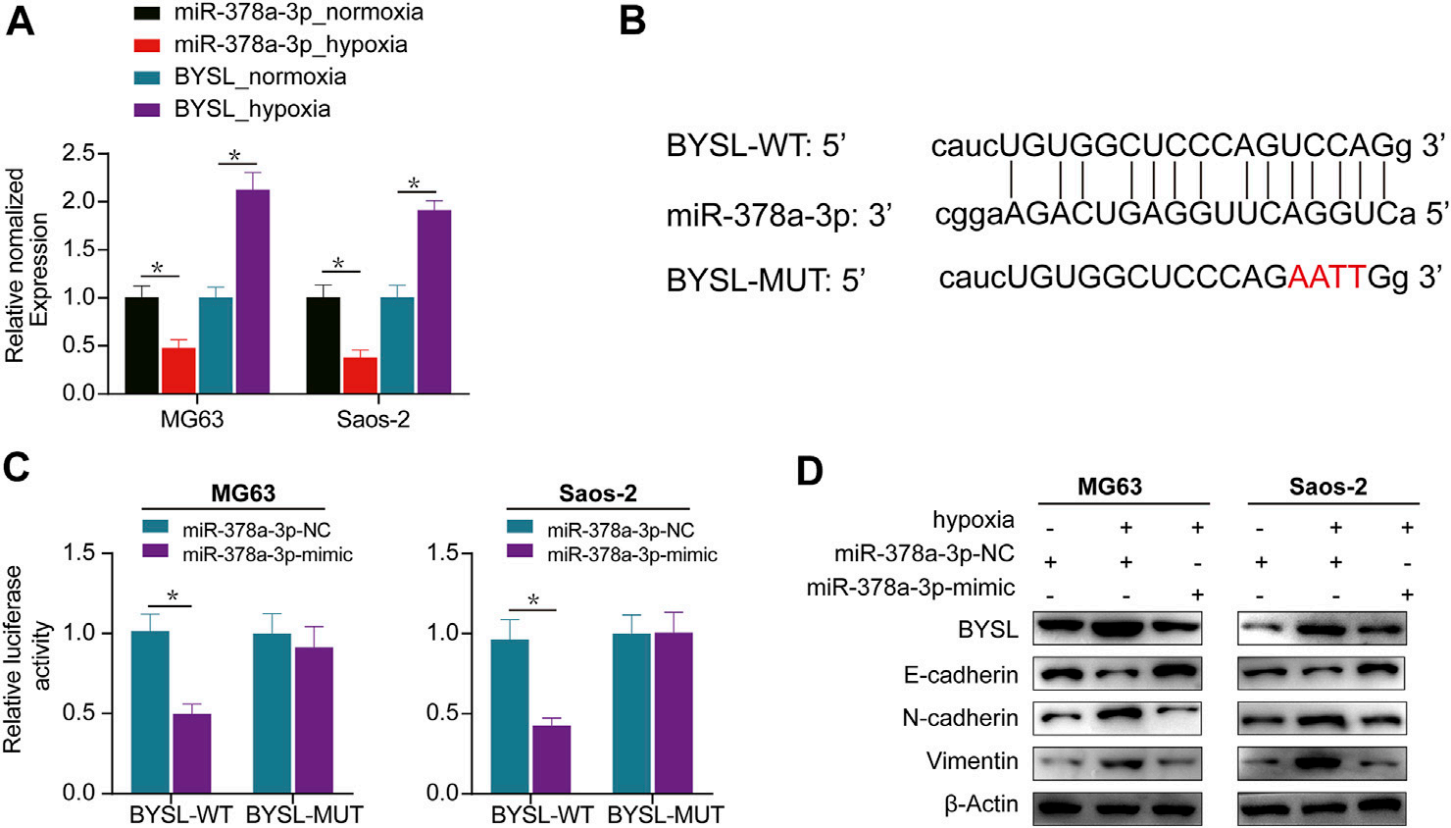
*BYSL* is a direct target of miR-378a-3p. **(A)** MG63 and Saos-2 cells were cultured under hypoxic or normoxic conditions. The RNA levels of miR-378a-3p and BYSL were measured by RT-qPCR. **(B)** The 3′-untranslated region (UTR) of *BYSL* harbor potential miR-378a-3p binding sites. **(C)** The luciferase activity displayed by the luciferase reporter constructs which contained wild-type (WT) or mutant (MUT) 3′-UTR of *BYSL* were co-transfected with miR-378a-3p mimic into MG63 and Saos-2 cells. **(D)** MG63 and Saos-2 cells were transfected with control-mimic (miR-378a-3p-NC) or miR-378a-3p-mimic, and then cultured under hypoxic or normoxic conditions. The protein levels of BYSL, E-cadherin, N-cadherin, and Vimentin were measured by western blot. The data are presented as the mean ± SD. *p < 0.05.

Next, we used miRTarBase and ENCORI databases to examine the potential *BYSL*-targeting microRNAs and predict the probable functional binding site. Among the candidate microRNAs, we were particularly interested in miR-378a-3p owing to its potential tumor suppressing effect in cancer development. Furthermore, qRT-PCR data showed that hypoxia increased *BYSL* expression and reduced that of miR-378a-3p, indicating *BYSL* correlated inversely with miR-378a-3p (Figure 2A). Based on these results, we tested whether *BYSL* could be a direct target of miR-378a-3p using a luciferase reporter assay. Cells were cotransfected with luciferase reporters containing *BYSL*-UTR-WT or *BYSL*-UTR-MUT along with miR-378a-3p-mimic. Transfection of miR-378a-3p-mimic markedly inhibited the luciferase reporter activity of WT but not of MUT (Figures 2B,C). Taken together, these results indicate the direct binding of miR-378a-3p to the 3′-UTR of *BYSL*. Next, we compared expression of BYSL and EMT markers by western blot under normoxia and hypoxia. Reduced expression of E-cadherin, and increased expressions of BYSL, vimentin, and N-cadherin were observed under hypoxia. Interestingly, miR-378a-3p overexpression rescued the effects of hypoxia on osteosarcoma cells (Figure 2D, Supplementary Figure S3), indicating that miR-378a-3p decreased hypoxia-induced BYSL expression to inhibit EMT.

### miR-378a-3p Knockdown Promotes EMT and Invasion by Elevating *BYSL* Expression Under Normoxia

In osteosarcoma cells, the expression of miR-378a-3p was downregulated after exposure to hypoxia compared to normoxia. Thus, we examined the role of miR-378a-3p in EMT using knockdown or overexpression assays under normoxia or hypoxia, respectively.

**FIGURE 3:**
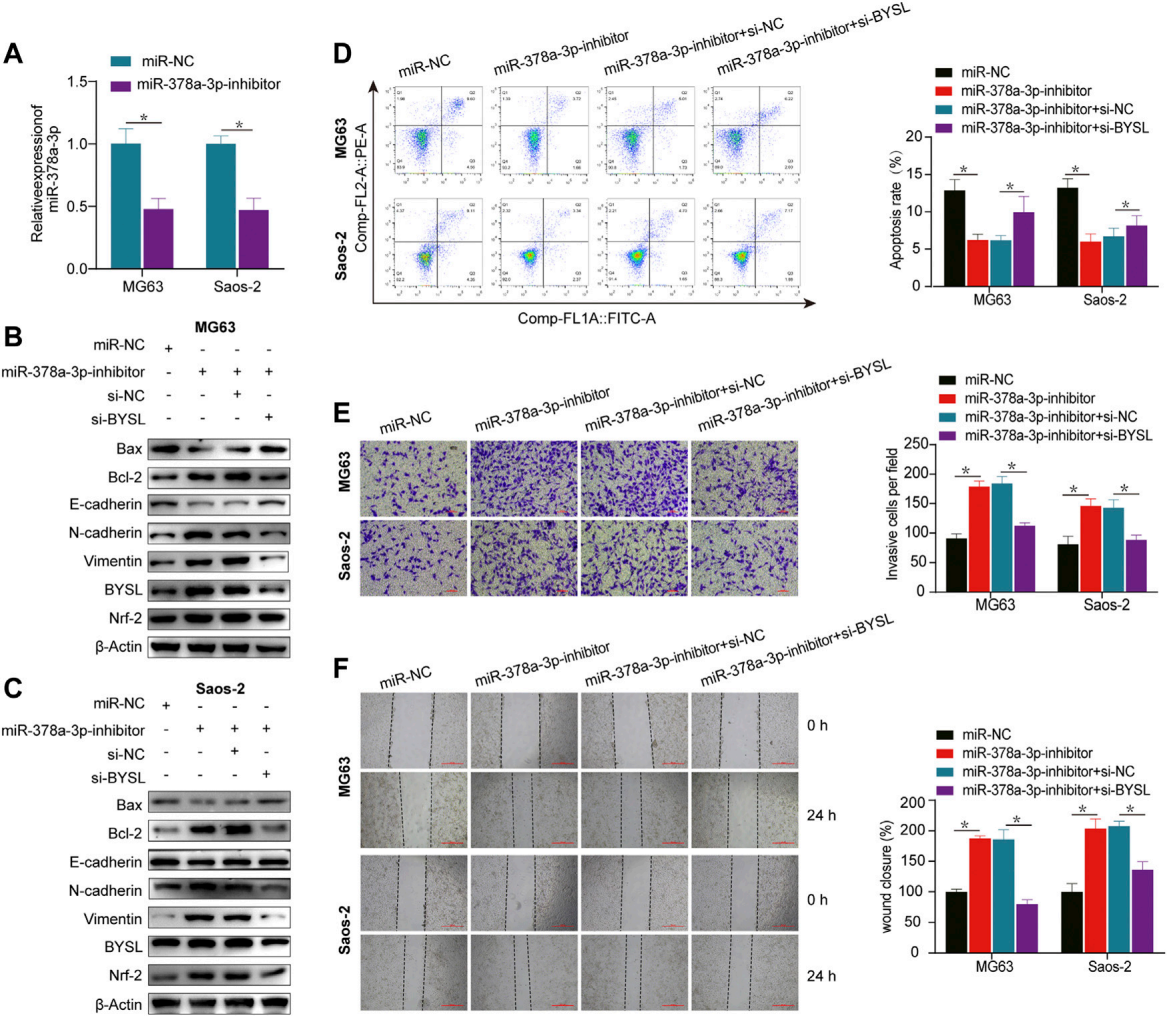
*BYSL* knockdown partially abolishes miR-378a-3p-mediated osteosarcoma cell epithelial-to-mesenchymal transition (EMT), invasion, migration, and apoptosis under normoxia. **(A)** MG63 and Saos-2 cells were transfected with control-inhibitor (miR-NC) or miR-378a-3p-inhibitor, and then cultured under normoxic conditions. The RNA level of miR-378a-3p was measured by RT-qPCR. **(B,C)** MG63 and Saos-2 cells were transfected with control-inhibitor (miR-NC), miR-378a-3p-inhibitor, si-control (si-NC), or si-BYSL, and then cultured under normoxic conditions. The protein levels of Bax, Bcl-2, E-cadherin, N-cadherin, vimentin, BYSL and Nrf2 were measured by western blot. **(D)** MG63 and Saos-2 cells were transfected with control-inhibitor (miR-NC), miR-378a-3p-inhibitor, si-control (si-NC), or si-BYSL, and then cultured under normoxic conditions. Cell apoptosis was measured by flow cytometry. **(E)** MG63 and Saos-2 cells were transfected with control-inhibitor (miR-NC), miR-378a-3p-inhibitor, si-control (si-NC), or si-BYSL, and then cultured under normoxic conditions. Cell invasion was measured by matrigel invasion assay. Scale bar = 100 µm. **(F)** MG63 and Saos-2 cells were transfected with control-inhibitor (miR-NC), miR-378a-3p-inhibitor, si-control (si-NC), or si-BYSL, and then cultured under normoxic conditions. Cell migration was measured by scratch wound healing assay. Scale bar = 500 µm. The data are presented as the mean ± SD. *p < 0.05.

qRT-PCR was performed to evaluate the transfection efficiency of miR-378a-3p, confirming that miR-378a-3p expression was significantly decreased in the knockdown group (Figure 3A). Under normoxic conditions, western blot analysis showed that miR-378a-3p knockdown resulted in reduced Bax and increased Bcl-2 expression, whereas *BYSL* silencing reversed this effect, suggesting that miR-378a-3p knockdown suppressed apoptosis (Figures 3B,C; Supplementary Figure S4). This outcome was further confirmed by flow cytometry (Figure 3D). Furthermore, miR-378a-3p knockdown promoted N-cadherin and vimentin expression and declined E-cadherin levels, whereas *BYSL* silencing reversed these effects (Figures 3B,C; Supplementary Figure S4). It was also observed that transfection of miR-378a-3p inhibitor increased BYSL and Nrf2 protein levels, whereas knockdown of BYSL attenuated the effect of miR-378a-3p on Nrf2 expression. We then performed matrigel invasion and wound healing assays to investigate whether miR-378a-3p could regulate the invasive and migratory abilities of osteosarcoma cells. Under normoxic conditions, miR-378a-3p knockdown greatly promoted the invasive (Figure 3E) and migratory potential (Figure 3F) of human osteosarcoma cells, and knockdown of *BYSL* rescued the miR-378a-3p-induced effects on osteosarcoma cells.

### miR-378a-3p Overexpression Inhibits EMT and Invasion by Suppressing *BYSL* Expression Under Hypoxia

**FIGURE 4:**
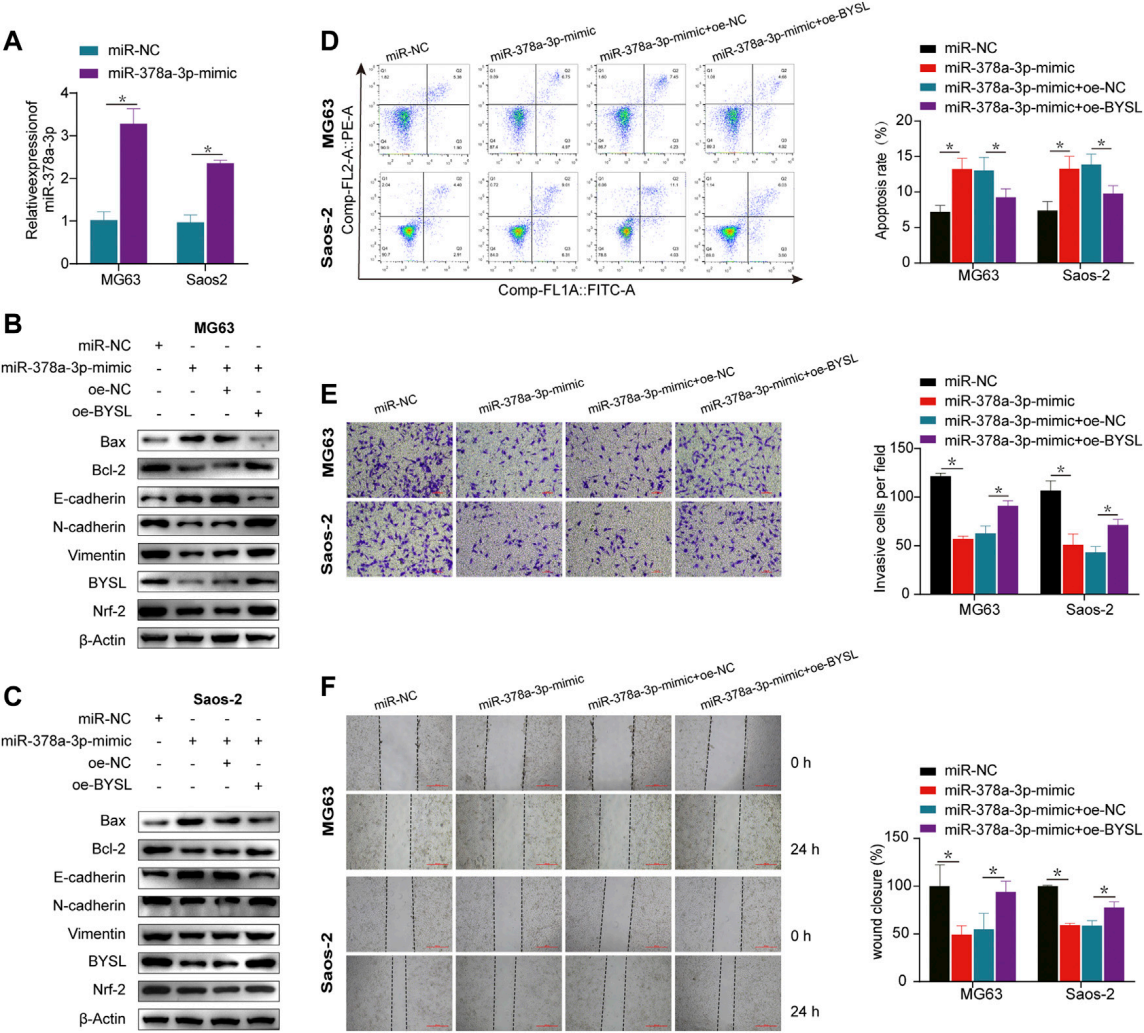
*BYSL* overexpression rescues the effect of miR-378a-3p overexpression on osteosarcoma cells under hypoxia. **(A)** MG63 and Saos-2 cells were transfected with control-mimic (miR-NC) or miR-378a-3p-mimic, and then cultured under hypoxic conditions. The RNA level of miR-378a-3p was measured by RT-qPCR. **(B,C)** MG63 and Saos-2 cells were transfected with control-mimic (miR-NC), miR-378a-3p-mimic, control plasmid (oe-NC), or BYSL overexpression plasmid (oe-BYSL), and then cultured under hypoxic conditions. The protein levels of Bax, Bcl-2, E-cadherin, N-cadherin, vimentin, BYSL and Nrf2 were measured by western blot. **(D)** MG63 and Saos-2 cells were transfected with control-mimic (miR-NC), miR-378a-3p-mimic, control plasmid (oe-NC), or BYSL overexpression plasmid (oe-BYSL), and then cultured under hypoxic conditions. Cell apoptosis was measured by flow cytometry. **(E)** MG63 and Saos-2 cells were transfected with control-mimic (miR-NC), miR-378a-3p-mimic, control plasmid (oe-NC), or BYSL overexpression plasmid (oe-BYSL), and then cultured under hypoxic conditions. Cell invasion was measured by matrigel invasion assay. Scale bar = 100 µm. **(F)** MG63 and Saos-2 cells were transfected with control-mimic (miR-NC), miR-378a-3p-mimic, control plasmid (oe-NC), or BYSL overexpression plasmid (oe-BYSL), and then cultured under hypoxic conditions. Cell migration was measured by scratch wound healing assay. Scale bar = 500 µm. The data are presented as the mean ± SD. *p < 0.05.

qRT-PCR was used to confirm that miR-378a-3p was significantly overexpressed in the overexpression group (Figure 4A). Western blot (Figures 4B,C; Supplementary Figure S5) and apoptosis analyses (Figure 4D) demonstrated that miR-378a-3p overexpression increased apoptosis in osteosarcoma cells under hypoxic conditions, which was inhibited by *BYSL* overexpression. Moreover, the protein levels of N-cadherin and vimentin were decreased, whereas E-cadherin was increased in miR-378a-3p-overexpressing cells. This phenotype can be reversed by *BYSL* overexpression (Figures 4B,C; Supplementary Figure S5). Osteosarcoma cells transfected with miR-378a-3p mimic had decreased BYSL and Nrf2 expression. However, overexpression of BYSL lead to an increase in Nrf2 expression, which was consistent with the above results (Figure 1E). The results of transwell assays (Figure 4E) and wound healing (Figure 4F) further demonstrated that miR-378a-3p overexpression inhibited osteosarcoma cell migration and invasion in hypoxia. However, upregulation of BYSL markedly attenuated the effect of miR-378a-3p on migration and invasion. In summary, these results indicate that miR-378a-3p regulates hypoxia-induced EMT and invasion by modulating *BYSL* expression.

### miR-378a-3p Overexpression Inhibits Osteosarcoma Cell Proliferation *In Vivo* and *In Vitro*

**FIGURE 5:**
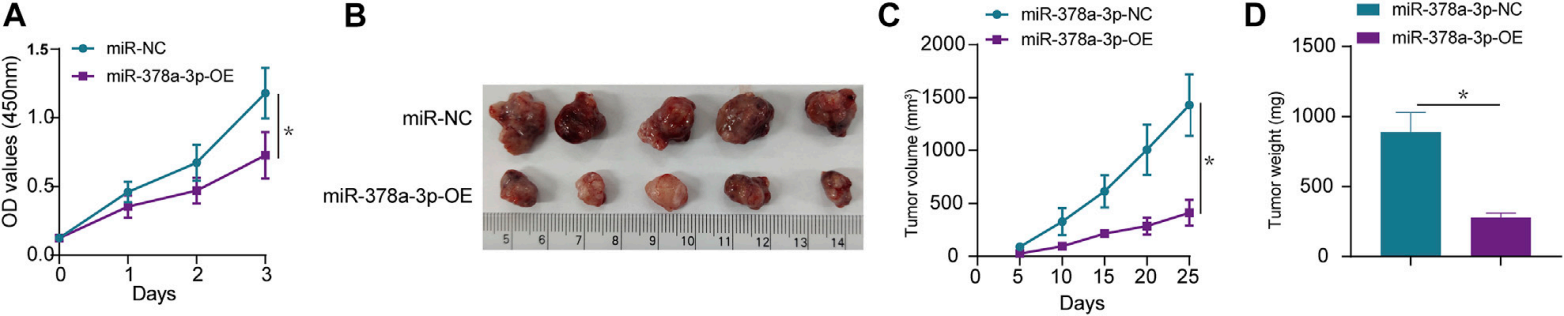
miR-378a-3p inhibits MG63 cell proliferation *in vivo* and *in vitro.* **(A)** MG63 cells were transfected with control-mimic (miR-NC), miR-378a-3p-mimic (miR-378a-3p-OE), and then cultured under hypoxic conditions. Cell proliferation assay using CCK8 assay. **(B–D)** Tumor weight and volume were examined after mice were sacrificed, representative tumors from corresponding treatments groups. (*n* = 5). The data are presented as the mean ± SD. *p < 0.05.

Subsequently, the *in vivo* experiments were conducted to further verify the function of miR-378a-3p in osteosarcoma. CCK-8 cell proliferation assay showed that in osteosarcoma cells, miR-378a-3p overexpression potently decreased CCK-8 optical density, indicating that miR-378a-3p suppressed osteosarcoma cell proliferation (Figure 5A). To verify the specificity of miR-378a-3p function in osteosarcoma tumors, an ectopic (subcutaneous) osteosarcoma tumor xenograft model was constructed (Figure 5B). Tumor size (Figure 5C) and weight (Figure 5D) showed that compared with the control group, osteosarcoma tumor growth was decreased in mice with tumors harboring stable miR-378a-3p overexpression. These data indicate that miR-378a-3p inhibits osteosarcoma tumor growth *in vivo*.

## Discussion

*BYSL* is an evolutionarily conserved gene that is expressed in a wide range of eukaryotes (Pack et al., 1998). It was initially identified as a cytoplasmic protein expressed in human trophoblastic embryonal carcinoma cells, forming a complex with theophanic and tastin (Suzuki et al., 1998; Fukuda and Nozawa, 1999). However, the function of *BYSL* in osteosarcoma remains poorly understood. Studies have shown that *BYSL* contributes to tumor cell growth and survival by forming a complex with mTORC2 in gliomas (Gao et al., 2021). Furthermore, high expression of *BYSL* in glioblastoma has been shown to strongly correlate with markers of mesenchymal glioblastoma (Sha et al., 2020). Herein, bioinformatics analysis of TCGA data showed that *BYSL* overexpression is associated with an unsatisfactory prognosis. The present study provides evidence that high *BYSL* expression is related to metastasis, TNM stage, and relapse. Moreover, survival analysis showed that patients with high *BYSL* expression have significantly worse overall survival than those with low expression.

EMT, a key developmental process, is frequently activated during embryonic morphogenesis and metastasis. In the present study, *BYSL* overexpression promoted EMT in MG63 and Saos-2 cells, indicating that *BYSL* may exert a critical effect on the invasive and aggressive behavior of osteosarcoma. Many evolutionarily conserved genes seem to correlate with diverse cellular functions during embryonic development and tumorigenesis. For example, an evolutionarily conserved metabolic gene, *LOX*, is involved in gliomagenesis (Chi et al., 2019). Fascin is an evolutionarily highly conserved protein that is involved in cell migration and tumor metastasis (Mattila and Lappalainen, 2008; Sun et al., 2011). Additionally, p53 family members, including p53, p63, and p73, are evolutionarily well conserved, and their main function is to maintain the genomic integrity of germ cells (Hu et al., 2011). A previous study also revealed that *BYSL* is essential for blastocyst formation. Mechanistically, blocking *BYSL* function causes defects in 40S ribosomal subunit biogenesis (Adachi et al., 2007). In the tumor microenvironment, hypoxia is an important hallmark that regulates angiogenesis and tumor growth (Hanahan and Weinberg, 2011). It can drive the EMT process through activation of HIF-1α signaling (Muz et al., 2015). Nrf2 is involved in the stabilization of the hybrid epithelial/mesenchymal phenotype in cancer cells (Bocci et al., 2019). Under hypoxia, HIF-1α signaling increases the expression of Nrf2, which interacts with TRX1, leading to enhanced HIF-1α expression (Toth and Warfel, 2017). Hypoxia activates Nrf2 signaling, which exerts a significant effect on the regulation of EMT in cancer cells (Bocci et al., 2019). Therefore, we investigated whether *BYSL* expression could affect Nrf2 signaling in MG63 and Saos-2 cells. Generally, stabilized Nrf2 migrates into the nucleus in a heterodimer with a small MAF (sMAF) transcription factor (Itoh et al., 1997), promoting cytoprotective genes transcription (Rushmore and Pickett, 1990). In the present study, we observed hypoxia stimulates Nrf2 nuclear translocation. Meanwhile, overexpression of BYSL increased Nrf2 expression and promoted EMT process under hypoxic conditions. Our rescue experiments revealed that knockdown of Nrf2 could reverse the effect of BYSL on EMT. It was thereby inferred, BYSL may modulate EMT by regulating Nrf2 signaling. Together, these findings suggest that *BYSL* is a pro-oncogenic protein and, thus, *BYSL* inhibition may represent a promising molecular therapeutic strategy for treating osteosarcoma in humans.

Several microRNAs have been shown to modulate various biological processes in osteosarcoma cells (Luo et al., 2020). For example, low expression of miR-1225-5p is correlated with poor prognosis in patients with osteosarcoma, and its overexpression inhibits osteosarcoma cell invasion and metastasis by targeting *Sox9* (Zhang et al., 2020). *In vivo* and *in vitro* studies have shown that miR-223-3p upregulation reduces osteosarcoma cell invasion, migration, growth, and proliferation by reducing *CDH6* expression (Ji et al., 2018). Liu et al. also reported that overexpression of miR-95-3p suppresses osteosarcoma cell growth by targeting *HDGF* (Liu et al., 2019). Moreover, miR-378a-3p inhibits the proliferation and migration of glioblastoma (Guo et al., 2019). In esophageal squamous cell carcinoma (ESCC), miR-378a-3p functions as a tumor suppressor to inhibit the migration, proliferation, and invasion of ESCC cells (Ding et al., 2018). However, whether miR-378a-3p participates in osteosarcoma progression remains unclear. In the present study, miR-378a-3p was identified as being downregulated in osteosarcoma cells under hypoxic conditions. Interestingly, miR-378a-3p has been linked to the modulation of ischemia/reperfusion kidney injury (Ding et al., 2020), suggesting that is implicated in hypoxia. Previous reports have shown that hypoxia induces EMT in various tumors (Lu et al., 2020; Xing et al., 2021). Similarly, we found that hypoxia contributed to EMT via miR378a-3p downregulation. Luciferase reporter assays and rescue experiments demonstrated that miR-378a-3p inhibits hypoxia-induced EMT, invasion, and migration of osteosarcoma cells by targeting *BYSL*. Moreover, results of *in vivo* experiments further demonstrated that miR-378a-3p overexpression inhibits osteosarcoma cell proliferation. Therefore, miR-378a-3p maybe associated with osteosarcoma metastasis. Futher clinical studies are required to understand the detailed functions of miR-378a-3p in osteosarcoma (Supplementary Figure S6).

In summary, the present study demonstrates that *BYSL* is an independent factor for evaluating the prognosis of patients with osteosarcoma. Additionally, miR-378a-3p can inhibit EMT and invasion by directly targeting *BYSL*. The miR-378a-3p/BYSL axis may play a role in osteosarcoma, and the cilinical significance of miR-378a-3p in osteosarcoma patients should be explored in the future.

## Data Availability

The datasets presented in this study can be found in online repositories. The names of the repository/repositories and accession number(s) can be found in the article/Supplementary Material.

## Supplementary Material

Supplementary Figure S1 Total BYSL, nuclear BYSL, Nrf2, and cytoplasmic Nrf2 in MG63 and Saos-2 were examined using western blot. **p* < 0.05.

Supplementary Figure S2 BYSL, Nrf2, E-cadherin, N-cadherin, and vimentin in MG63 and Saos-2 under hypoxia were examined using western blot. **p* < 0.05.

Supplementary Figure S3 BYSL, E-cadherin, N-cadherin, and vimentin in MG63 and Saos-2 were examined using western blot. **p* < 0.05.

Supplementary Figure S4 Bax, Bcl2, E-cadherin, N-cadherin, and vimentin, BYSL, and Nrf2 were examined using western blot under normoxia. **p* < 0.05.

Supplementary Figure S5 Bax, Bcl2, E-cadherin, N-cadherin, and vimentin, BYSL, and Nrf2 were examined using western blot under hypoxia. **p* < 0.05.

Supplementary Figure S6 Overall survival was compared between osteosarcoma patients with high and low *miR-378a-3p* expression in TCGA database.

## References

[B1] Adachi K. Soeta-Saneyoshi C. Sagara H. Iwakura Y. (2007). Crucial Role of Bysl in Mammalian Preimplantation Development as an Integral Factor for 40S Ribosome Biogenesis. Mol. Cel. Biol. 27 (6), 2202–2214. 10.1128/mcb.01908-06 PMC1820511 17242206

[B2] Ando K. Heymann M.-F. Stresing V. Mori K. Rédini F. Heymann D. (2013). Current Therapeutic Strategies and Novel Approaches in Osteosarcoma. Cancers 5 (2), 591–616. 10.3390/cancers5020591 24216993 PMC3730336

[B3] Aoki R. Suzuki N. Paria B. C. Sugihara K. Akama T. O. Raab G. (2006). TheByslgene Product, Bystin, Is Essential for Survival of Mouse Embryos. FEBS Lett. 580 (26), 6062–6068. 10.1016/j.febslet.2006.09.072 17055491 PMC1764500

[B4] Arias A. M. (2001). Epithelial Mesenchymal Interactions in Cancer and Development. Cell 105 (4), 425–431. 10.1016/s0092-8674(01)00365-8 11371340

[B5] Bartel D. P. (2004). MicroRNAs. Cell 116 (2), 281–297. 10.1016/s0092-8674(04)00045-5 14744438

[B6] Bocci F. Tripathi S. C. Vilchez Mercedes S. A. George J. T. Casabar J. P. Wong P. K. (2019). NRF2 Activates a Partial Epithelial-Mesenchymal Transition and Is Maximally Present in a Hybrid Epithelial/mesenchymal Phenotype. Integr. Biol. (Camb) 11 (6), 251–263. 10.1093/intbio/zyz021 31329868 PMC6686740

[B7] Buddingh E. P. Kuijjer M. L. Duim R. A. J. Bürger H. Agelopoulos K. Myklebost O. (2011). Tumor-infiltrating Macrophages Are Associated with Metastasis Suppression in High-Grade Osteosarcoma: a Rationale for Treatment with Macrophage Activating Agents. Clin. Cancer Res. 17 (8), 2110–2119. 10.1158/1078-0432.ccr-10-2047 21372215

[B8] Chanjiao Y. Chunyan C. Xiaoxin Q. Youjian H. (2021). MicroRNA‐378a‐3p Contributes to Ovarian Cancer Progression through Downregulating PDIA4. Immun. Inflamm. Dis. 9 (1), 108–119. 10.1002/iid3.350 33159506 PMC7860521

[B9] Chi K.-C. Tsai W.-C. Wu C.-L. Lin T.-Y. Hueng D.-Y. (2019). An Adult Drosophila Glioma Model for Studying Pathometabolic Pathways of Gliomagenesis. Mol. Neurobiol. 56 (6), 4589–4599. 10.1007/s12035-018-1392-2 30357574

[B10] Ding C. Ding X. Zheng J. Wang B. Li Y. Xiang H. (2020). miR-182-5p and miR-378a-3p Regulate Ferroptosis in I/R-induced Renal Injury. Cell Death Dis. 11 (10), 929. 10.1038/s41419-020-03135-z 33116120 PMC7595188

[B11] Ding N. Sun X. Wang T. Huang L. Wen J. Zhou Y. (2018). miR-378a-3p Exerts Tumor Suppressive Function on the Tumorigenesis of Esophageal Squamous Cell Carcinoma by Targeting Rab10. Int. J. Mol. Med. 42 (1), 381–391. 10.3892/ijmm.2018.3639 29693138 PMC5979826

[B12] Fukuda M. Nozawa S. (1999). Trophinin, Tastin, and Bystin: a Complex Mediating Unique Attachment between Trophoblastic and Endometrial Epithelial Cells at Their Respective Apical Cell Membranes. Semin. Reprod. Med. 17 (3), 229–234. 10.1055/s-2007-1016230 10797941

[B13] Gao S. Sha Z. Zhou J. Wu Y. Song Y. Li C. (2021). BYSL Contributes to Tumor Growth by Cooperating with the mTORC2 Complex in Gliomas. Cancer Biol. Med. 18 (1), 88–104. 10.20892/j.issn.2095-3941.2020.0096 33628587 PMC7877178

[B14] Gorlick S. Barkauskas D. A. Krailo M. Piperdi S. Sowers R. Gill J. (2014). HER-2 Expression Is Not Prognostic in Osteosarcoma; a Children's Oncology Group Prospective Biology Study. Pediatr. Blood Cancer 61 (9), 1558–1564. 10.1002/pbc.25074 24753182 PMC4288578

[B15] Guo X. B. Zhang X. C. Chen P. Ma L. M. Shen Z. Q. (2019). miR-378a-3p Inhibits Cellular Proliferation and Migration in Glioblastoma Multiforme by Targeting Tetraspanin 17. Oncol. Rep. 42 (5), 1957–1971. 10.3892/or.2019.7283 31432186 PMC6775804

[B16] Hanahan D. Weinberg R. A. (2011). Hallmarks of Cancer: the Next Generation. Cell 144 (5), 646–674. 10.1016/j.cell.2011.02.013 21376230

[B17] Harrison D. J. Geller D. S. Gill J. D. Lewis V. O. Gorlick R. (2018). Current and Future Therapeutic Approaches for Osteosarcoma. Expert Rev. Anticancer Ther. 18 (1), 39–50. 10.1080/14737140.2018.1413939 29210294

[B18] Hu W. Zheng T. Wang J. (2011). Regulation of Fertility by the P53 Family Members. Genes & Cancer 2 (4), 420–430. 10.1177/1947601911408892 21779510 PMC3135638

[B19] Ikeda K. Horie-Inoue K. Ueno T. Suzuki T. Sato W. Shigekawa T. (2015). miR-378a-3p Modulates Tamoxifen Sensitivity in Breast Cancer MCF-7 Cells through Targeting GOLT1A. Sci. Rep. 5, 13170. 10.1038/srep13170 26255816 PMC4530347

[B20] Itoh K. Chiba T. Takahashi S. Ishii T. Igarashi K. Katoh Y. (1997). An Nrf2/small Maf Heterodimer Mediates the Induction of Phase II Detoxifying Enzyme Genes through Antioxidant Response Elements. Biochem. Biophysical Res. Commun. 236 (2), 313–322. 10.1006/bbrc.1997.6943 9240432

[B21] Ji Q. Xu X. Song Q. Xu Y. Tai Y. Goodman S. B. (2018). miR-223-3p Inhibits Human Osteosarcoma Metastasis and Progression by Directly Targeting CDH6. Mol. Ther. 26 (5), 1299–1312. 10.1016/j.ymthe.2018.03.009 29628305 PMC5993963

[B22] Joseph J. P. Harishankar M. K. Pillai A. A. Devi A. (2018). Hypoxia Induced EMT: A Review on the Mechanism of Tumor Progression and Metastasis in OSCC. Oral Oncol. 80, 23–32. 10.1016/j.oraloncology.2018.03.004 29706185

[B23] Lee Y. S. Dutta A. (2009). MicroRNAs in Cancer. Annu. Rev. Pathol. Mech. Dis. 4, 199–227. 10.1146/annurev.pathol.4.110807.092222 PMC2769253 18817506

[B24] Li J. Qin B. Huang M. Ma Y. Li D. Li W. (2021). Tumor-Associated Antigens (TAAs) for the Serological Diagnosis of Osteosarcoma. Front. Immunol. 12, 665106. 10.3389/fimmu.2021.665106 33995397 PMC8119874

[B25] Liu M. Si Q. Ouyang S. Zhou Z. Wang M. Zhao C. (2020). Serum MiR-4687-3p Has Potential for Diagnosis and Carcinogenesis in Non-small Cell Lung Cancer. Front. Genet. 11, 597508. 10.3389/fgene.2020.597508 33329742 PMC7721467

[B26] Liu X. Ma W. Ma J. Xiao L. Hao D. (2019). Upregulation of miR-95-3p I-nhibits G-rowth of O-steosarcoma by T-argeting HDGF. Pathol. - Res. Pract. 215 (8), 152492. 10.1016/j.prp.2019.152492 31257090

[B27] Lu Y. Liu Y. Oeck S. Zhang G. J. Schramm A. Glazer P. M. (2020). Hypoxia Induces Resistance to EGFR Inhibitors in Lung Cancer Cells via Upregulation of FGFR1 and the MAPK Pathway. Cancer Res. 80 (21), 4655–4667. 10.1158/0008-5472.can-20-1192 32873635 PMC7642024

[B28] Luo H. Wang P. Ye H. Shi J. Dai L. Wang X. (2020). Serum-Derived microRNAs as Prognostic Biomarkers in Osteosarcoma: A Meta-Analysis. Front. Genet. 11, 789. 10.3389/fgene.2020.00789 32849795 PMC7431663

[B29] Mattila P. K. Lappalainen P. (2008). Filopodia: Molecular Architecture and Cellular Functions. Nat. Rev. Mol. Cel. Biol. 9 (6), 446–454. 10.1038/nrm2406 18464790

[B30] Muz B. de la Puente P. Azab F. Azab A. K. (2015). The Role of Hypoxia in Cancer Progression, Angiogenesis, Metastasis, and Resistance to Therapy. Hp 3, 83–92. 10.2147/hp.s93413 PMC5045092 27774485

[B31] Olczak M. Chutorański D. Kwiatkowska M. Samojłowicz D. Tarka S. Wierzba-Bobrowicz T. (2018). Bystin (BYSL) as a Possible Marker of Severe Hypoxic-Ischemic Changes in Neuropathological Examination of Forensic Cases. Forensic Sci. Med. Pathol. 14 (1), 26–30. 10.1007/s12024-017-9942-x 29349722 PMC5830468

[B32] Pack S. D. Pak E. Tanigami A. Ledbetter D. H. Fukuda M. N. (1998). Assignment1 of the Bystin Gene BYSL to Human Chromosome Band 6p21.1 by *In Situ* Hybridization. Cytogenet. Genome Res. 83 (1-2), 76–77. 10.1159/000015131 9925933

[B33] Pan X. Zhao L. Quan J. Liu K. Lai Y. Li Z. (2019). MiR-378a-5p Acts as a Tumor Suppressor in Renal Cell Carcinoma and Is Associated with the Good Prognosis of Patients. Am. J. Transl. Res. 11 (4), 2207–2218. 31105829 PMC6511777

[B34] Raymond A. K. Jaffe N. (2009). Osteosarcoma Multidisciplinary Approach to the Management from the Pathologist's Perspective. Cancer Treat. Res. 152, 63–84. 10.1007/978-1-4419-0284-9_4 20213386

[B35] Rushmore T. H. Pickett C. B. (1990). Transcriptional Regulation of the Rat Glutathione S-Transferase Ya Subunit Gene. Characterization of a Xenobiotic-Responsive Element Controlling Inducible Expression by Phenolic Antioxidants. J. Biol. Chem. 265 (24), 14648–14653. 10.1016/s0021-9258(18)77351-1 2387873

[B36] Sevelda F. Mayr L. Kubista B. Lötsch D. van Schoonhoven S. Windhager R. (2015). EGFR Is Not a Major Driver for Osteosarcoma Cell Growth *In Vitro* but Contributes to Starvation and Chemotherapy Resistance. J. Exp. Clin. Cancer Res. 34, 134. 10.1186/s13046-015-0251-5 26526352 PMC4630894

[B37] Sha Z. Zhou J. Wu Y. Zhang T. Li C. Meng Q. (2020). BYSL Promotes Glioblastoma Cell Migration, Invasion, and Mesenchymal Transition through the GSK-3β/β-Catenin Signaling Pathway. Front. Oncol. 10, 565225. 10.3389/fonc.2020.565225 33178594 PMC7593785

[B38] Shi C. Huang C.-M. Wang B. Sun T.-F. Zhu A.-X. Zhu Y.-C. (2020). Pseudogene MSTO2P Enhances Hypoxia-Induced Osteosarcoma Malignancy by Upregulating PD-L1. Biochem. Biophysical Res. Commun. 530 (4), 673–679. 10.1016/j.bbrc.2020.07.113 32768186

[B39] Sun J. He H. Xiong Y. Lu S. Shen J. Cheng A. (2011). Fascin Protein Is Critical for Transforming Growth Factor β Protein-Induced Invasion and Filopodia Formation in Spindle-Shaped Tumor Cells. J. Biol. Chem. 286 (45), 38865–38875. 10.1074/jbc.M111.270413 21914811 PMC3234711

[B40] Suzuki N. Zara J. Sato T. Ong E. Bakhiet N. Oshima R. G. (1998). A Cytoplasmic Protein, Bystin, Interacts with Trophinin, Tastin, and Cytokeratin and May Be Involved in Trophinin-Mediated Cell Adhesion between Trophoblast and Endometrial Epithelial Cells. Proc. Natl. Acad. Sci. 95 (9), 5027–5032. 10.1073/pnas.95.9.5027 9560222 PMC20207

[B41] Toth R. Warfel N. (2017). Strange Bedfellows: Nuclear Factor, Erythroid 2-Like 2 (Nrf2) and Hypoxia-Inducible Factor 1 (HIF-1) in Tumor Hypoxia. Antioxidants 6 (2), 27. 10.3390/antiox6020027 PMC5488007 28383481

[B42] Wang H. Xiao W. Zhou Q. Chen Y. Yang S. Sheng J. (2009). Bystin-like Protein Is Upregulated in Hepatocellular Carcinoma and Required for Nucleologenesis in Cancer Cell Proliferation. Cell Res. 19 (10), 1150–1164. 10.1038/cr.2009.99 19687802

[B43] Wang S. Zhang D. Han S. Gao P. Liu C. Li J. (2017). Fibulin-3 Promotes Osteosarcoma Invasion and Metastasis by Inducing Epithelial to Mesenchymal Transition and Activating the Wnt/β-Catenin Signaling Pathway. Sci. Rep. 7 (1), 6215. 10.1038/s41598-017-06353-2 28740094 PMC5524709

[B44] Xing S. Tian Z. Zheng W. Yang W. Du N. Gu Y. (2021). Hypoxia Downregulated miR-4521 Suppresses Gastric Carcinoma Progression through Regulation of IGF2 and FOXM1. Mol. Cancer 20 (1), 9. 10.1186/s12943-020-01295-2 33407516 PMC7786912

[B45] Zhang W. Wei L. Sheng W. Kang B. Wang D. Zeng H. (2020). miR-1225-5p Functions as a Tumor Suppressor in Osteosarcoma by Targeting Sox9. DNA Cel Biol. 39 (1), 78–91. 10.1089/dna.2019.5105 31765229

[B46] Zheng S. Li M. Miao K. Xu H. (2020). lncRNA GAS5‐promoted Apoptosis in Triple‐negative Breast Cancer by Targeting miR‐378a‐5p/SUFU Signaling. J. Cel. Biochem. 121 (3), 2225–2235. 10.1002/jcb.29445 31692053

