# Supplementary figures and images for: Hypoxia-Induced miR-378a-3p Inhibits Osteosarcoma Invasion and Epithelial-to-Mesenchymal Transition via BYSL Regulation
Source: Front Genet. 2022 Jan 28;12:804952. doi: 10.3389/fgene.2021.804952 (PMC8831866; doi:10.3389/fgene.2021.804952)

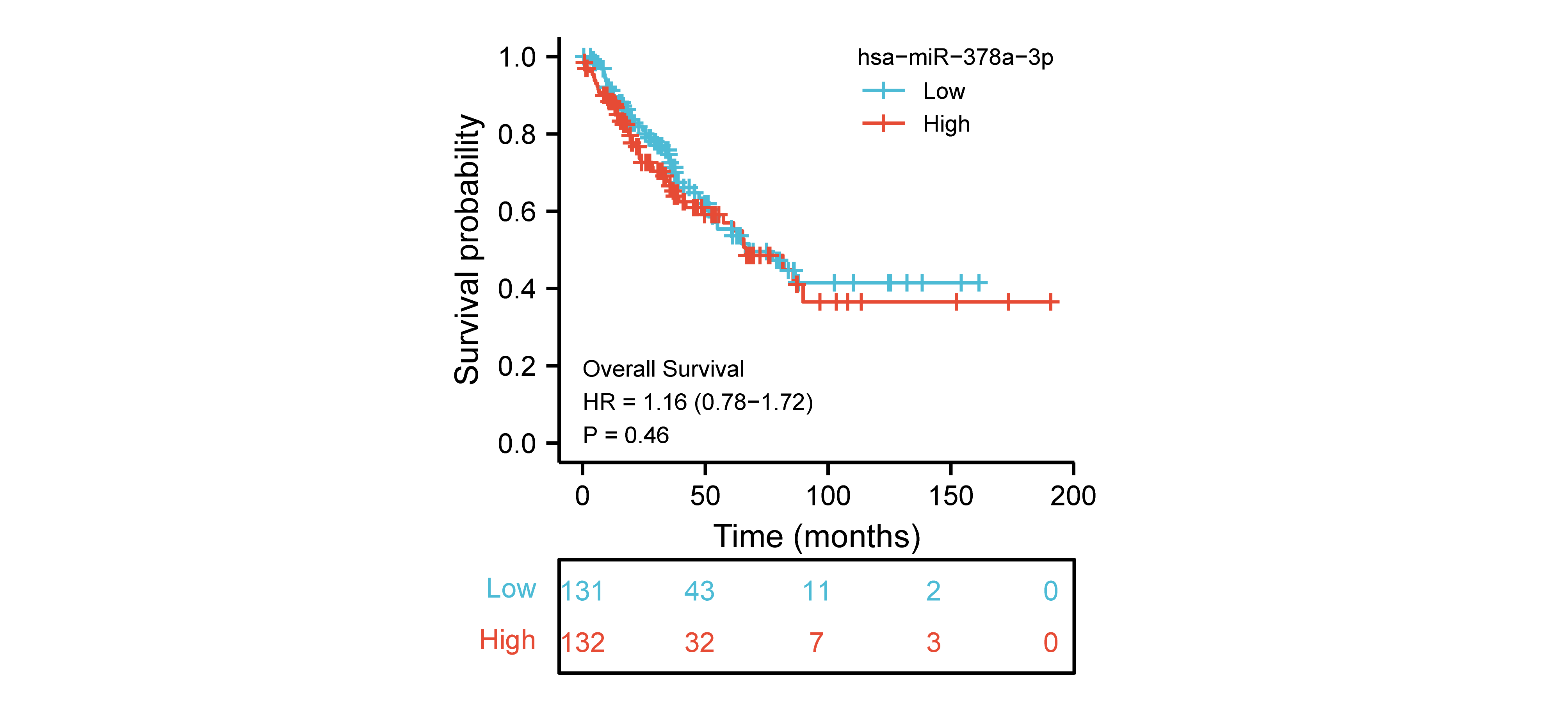

Supplement: Supplementary file 1 [file Image6.TIF]

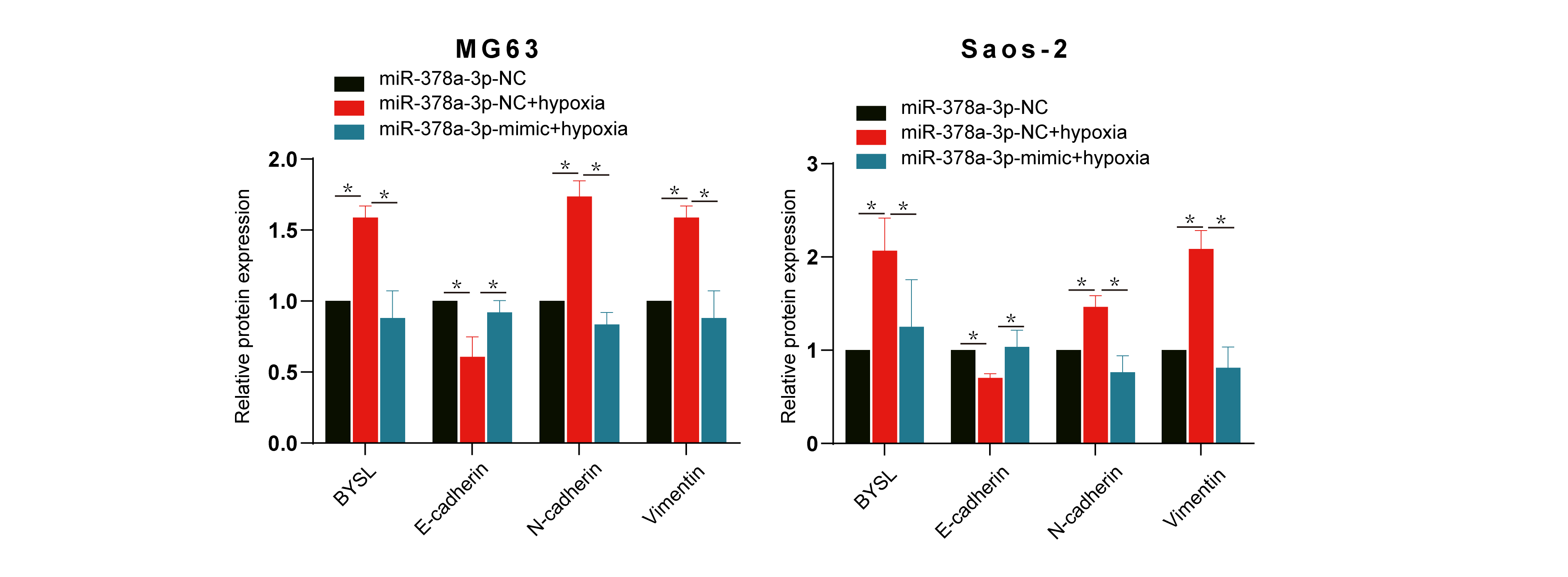

Supplement: Supplementary file 3 [file Image3.TIF]

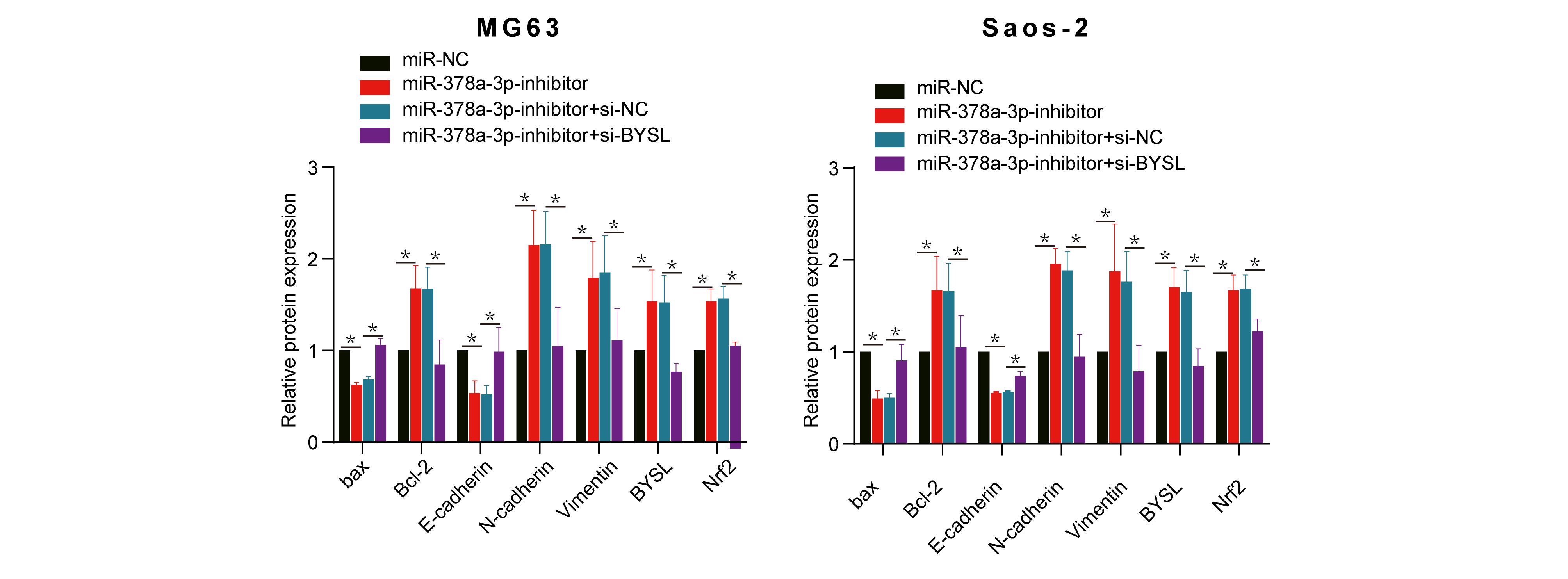

Supplement: Supplementary file 4 [file Image4.TIF]

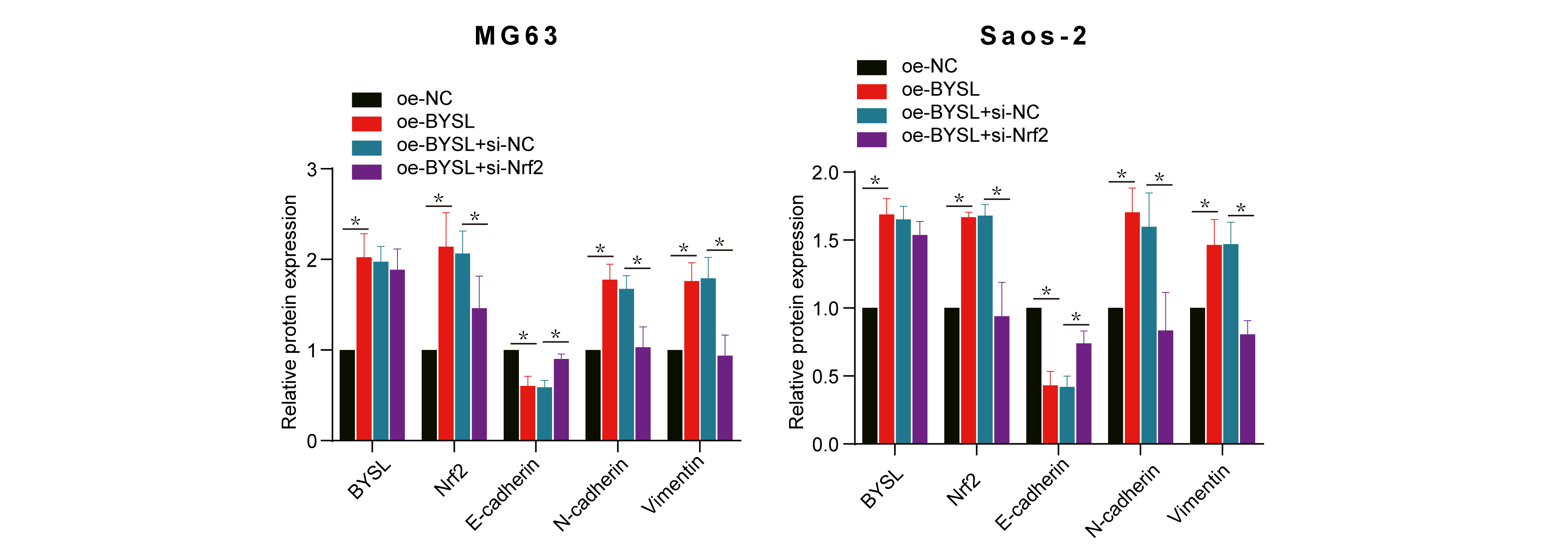

Supplement: Supplementary file 5 [file Image2.TIF]

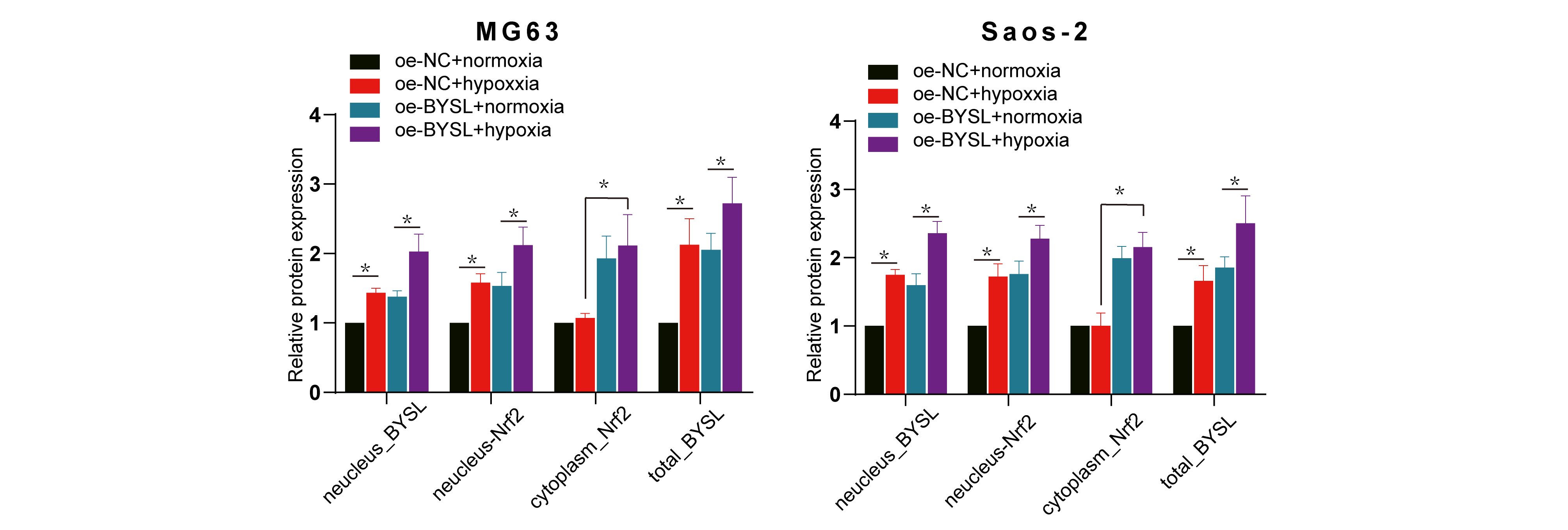

Supplement: Supplementary file 6 [file Image1.TIF]

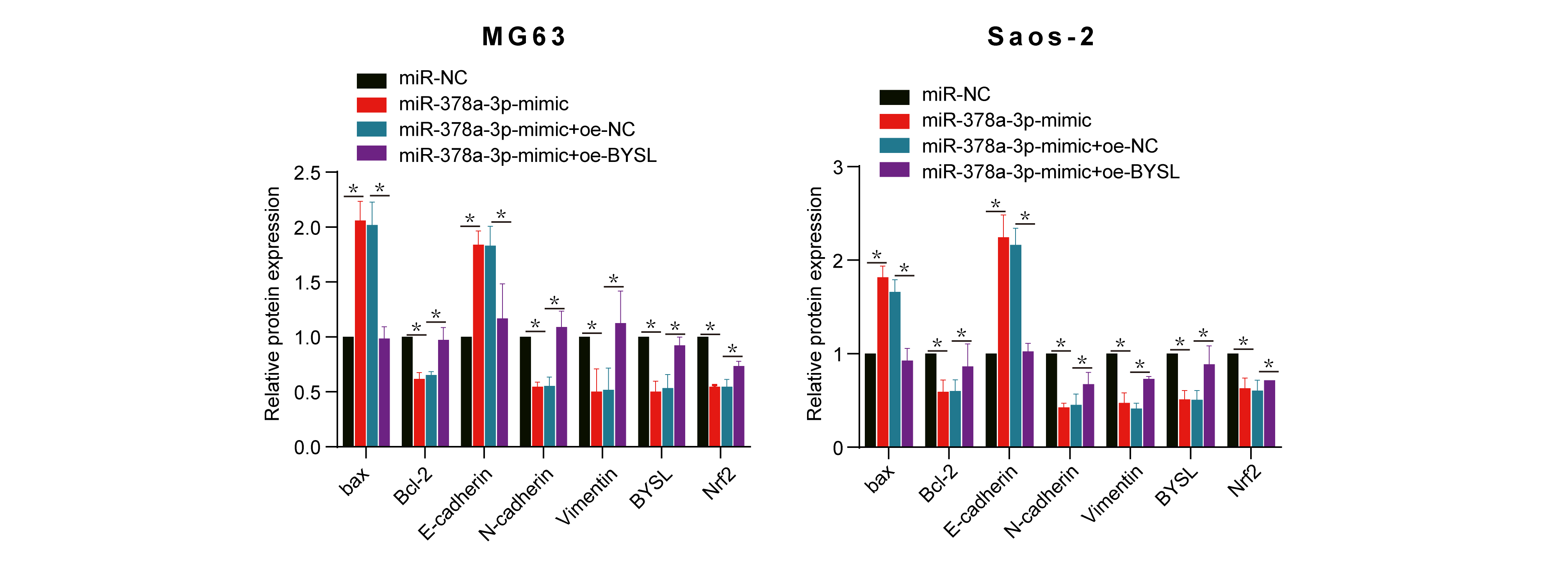

Supplement: Supplementary file 7 [file Image5.TIF]
