# Supplementary material for: Hypoxia-Induced miR-378a-3p Inhibits Osteosarcoma Invasion and Epithelial-to-Mesenchymal Transition via BYSL Regulation
Source: Front Genet. 2022 Jan 28;12:804952. doi: 10.3389/fgene.2021.804952 (PMC8831866; doi:10.3389/fgene.2021.804952)
Supplement: Supplementary file 2 [file Table1.DOCX]

**Table S1** RT-PCR primers of mRNAs in this study.

| mRNA | | | Forward(5’-3’) | Reverse(3’-5’) |
| --- | --- | --- | --- | --- |
| miR-378a-3p  BYSL | | CTCCTGACTCCAGGTCCTG  GTCTGGCACTTGTACCCTCC | | CAGTGCGTGTCGTGGAGT  TTGGCACCGCTGTATTCCAT |
| U6 | CTCGCTTCGGCAGCACA | | | AACGCTTCACGAATTTGCGT |
| GAPDH | | AGAAGGCTGGGGCTCATTTG | | AGGGGCCATCCACAGTCTTC |
